# Low-Frequency Repetitive Transcranial Magnetic Stimulation for Stroke-Induced Upper Limb Motor Deficit: A Meta-Analysis

**DOI:** 10.1155/2017/2758097

**Published:** 2017-12-21

**Authors:** Lan Zhang, Guoqiang Xing, Shiquan Shuai, Zhiwei Guo, Huaping Chen, Morgan A. McClure, Xiaojuan Chen, Qiwen Mu

**Affiliations:** ^1^Department of Imaging & Imaging Institute of Rehabilitation and Development of Brain Function, The Second Clinical Medical College of North Sichuan Medical College, Nanchong Central Hospital, Nanchong 637000, China; ^2^Department of Radiology, Langzhong People's Hospital, Nanchong 637000, China; ^3^Lotus Biotech.com LLC., John Hopkins University-MCC, Rockville, MD 20850, USA; ^4^North Sichuan Medical College, Nanchong 637000, China; ^5^Peking University Third Hospital, Beijing 100080, China

## Abstract

**Background and Purpose:**

This meta-analysis aimed to evaluate the therapeutic potential of low-frequency repetitive transcranial magnetic stimulation (LF-rTMS) over the contralesional hemisphere on upper limb motor recovery and cortex plasticity after stroke.

**Methods:**

Databases of PubMed, Medline, ScienceDirect, Cochrane, and Embase were searched for randomized controlled trials published before Jun 31, 2017. The effect size was evaluated by using the standardized mean difference (SMD) and a 95% confidence interval (CI). Resting motor threshold (rMT) and motor-evoked potential (MEP) were also examined.

**Results:**

Twenty-two studies of 1 Hz LF-rTMS over the contralesional hemisphere were included. Significant efficacy was found on finger flexibility (SMD = 0.75), hand strength (SMD = 0.49), and activity dexterity (SMD = 0.32), but not on body function (SMD = 0.29). The positive changes of rMT (SMD = 0.38 for the affected hemisphere and SMD = −0.83 for the unaffected hemisphere) and MEP (SMD = −1.00 for the affected hemisphere and SMD = 0.57 for the unaffected hemisphere) were also significant.

**Conclusions:**

LF-rTMS as an add-on therapy significantly improved upper limb functional recovery especially the hand after stroke, probably through rebalanced cortical excitability of both hemispheres. Future studies should determine if LF-rTMS alone or in conjunction with practice/training would be more effective.

**Clinical Trial Registration Information:**

This trial is registered with unique identifier CRD42016042181.

## 1. Introduction

Stroke is a global disease with high rates of long-term disability [1]. Around the world, 25%–74% of stroke survivors require different levels of assistance for daily living mainly due to upper limb hemiplegia [2]. In search for better therapies, scientists have been trying to understand the relationship between stroke motor recovery and cortical reorganization [3]. The equilibrium of cortical excitability between the two hemispheres is often disrupted after stroke. In the affected hemisphere, both the cortical excitability and the homonymous motor representation of the affected hemisphere decrease; whereas the excitability in the unaffected hemisphere increases [4].

Repetitive transcranial magnetic stimulation (rTMS) is a noninvasive stimulation to induce electrical currents in the brain tissues. Currently, rTMS is being explored as a novel therapy in modulating cortical excitability to improve motor functions in stroke patients [5]. Of the two forms of rTMS, high-frequency rTMS (HF-rTMS > 1.0 Hz), applied over the ipsilesional hemisphere, facilitates cortical excitability [6], whereas, low-frequency rTMS (LF-rTMS ≤ 1.0 Hz), applied over the contralesional hemisphere, decreases cortical excitability [7].

The effect of rTMS is primarily determined by the stimulation frequency [8] and targeted region [3]. Although both LF-rTMS and HF-rTMS could treat motor dysfunction in poststroke patients, LF-rTMS is considered safer and superior to HF-rTMS in motor function recovery [9–12]. Lomarev et al. [13] reported increased risk for seizures by HF-rTMS of 20–25 Hz. To date, the majority of rTMS trials on motor recovery after stroke used the protocol of LF-rTMS with 1 Hz. In comparison, the HF-rTMS studies involved only a small number of trials and applied varied frequency protocols (3 Hz to 25 Hz). According to Cho et al. [14], the primary motor cortex (M1) forms a main part of the motor cortices and contributes to the high order control of motor behaviors. Until now, most studies about the efficacy of LF-rTMS on functional rehabilitation have focused on the M1. In healthy subjects, LF-rTMS applied over the M1 increased the resting motor threshold (rMT) and decreased the motor-evoked potential (MEP) size of the ipsilateral hemisphere, suggesting a suppressive effect of LF-rTMS in the intact M1 [15].

Multiple studies have investigated the therapeutic effect of LF-rTMS after stroke [8, 16–19], with the outcomes of pinch force [19–22], grip force [10, 22–25], finger tapping [8, 9, 26–29], and overall function [15, 30–34]. Other studies also explored the impact of rTMS on cortical excitability [10, 18, 19, 26]. However, inconsistent reports exist regarding the benefits of LF-rTMS: Some studies showed no beneficial effect of LF-rTMS [16, 23, 29] and one study reported worsening effects of LF-rTMS such as decreased finger-tapping speed; [35] other investigators proposed that inhibition of the contralesional motor areas may lead to deterioration of the function of the unaffected hand [24, 26]. Although a few previous meta-analyses had investigated the therapeutic effect of rTMS after stroke [11, 36–38], they focused on the mixed effect of combined LF-rTMS and HF-rTMS interventions or on the combined outcomes of varying motor measurements. So far, there is a lack of in-depth systematic meta-analysis about the efficacy of LF-rTMS on upper limb function recovery.

The primary objective of this study was to evaluate the effects of LF-rTMS on upper limb motor recovery after stroke in several aspects: “finger flexibility,” “hand strength,” “activity dexterity,” and “body function level.” The effects of LF-rTMS on motor cortex excitability which were represented by MEP and rMTin poststroke patients were also evaluated.

## 2. Methods

### 2.1. Protocol

Our meta-analysis followed the PRISMA statement.

### 2.2. Search Strategy

The databases of PubMed, ScienceDirect, Embase, and the Cochrane Library were searched for randomized controlled trials published before June 31, 2017. The search terms were “stroke/cerebrovascular accident, repetitive transcranial magnetic stimulation/rTMS, and upper limb/hand.” The search was limited to human studies. Manual searches of the reference lists of the pertinent articles were also conducted to identify relevant articles [11, 36].

### 2.3. Study Selection

The preliminary screening was based on the title and abstract. As there were several separate aims of the paper, the articles with either any motor function assessment or MEP/rMT outcomes were all considered. Two reviewers independently assessed the eligibility of the literature. If there was a disagreement, the two reviewers checked the full text of the article and discussed with each other to reach an agreement. The selected articles were then assessed in their entirety. Studies were included if they met the following criteria: (1) they were randomized controlled trials; (2) they have ≥five patients in a trial; (3) the patients were adults (≥18 yrs); (4) the focus was on the effects on the upper limb in poststroke patients; (5) the types of intervention were LF-rTMS over the contralesional M1; (6) the outcomes were on continuous scales that evaluated the motor function of upper limb or cortical excitability; and (7) they were published in peer-reviewed English journals.

### 2.4. Quality Appraisal

Each included study was individually assessed by two reviewers according to a modified checklist of Moher et al. [39] that provided the following criteria: (1) blinding procedure (0 indicated a nonblind or no-mention procedure, 1 or 2 represented single blind or double blind, resp.); (2) dropout number; (3) description of baseline demographic data (was recorded as 1 if described, if not as 0); (4) point estimate and variability (was denoted as 1 if provided); and (5) description of adverse events (was recorded as the number and type of adverse event).

### 2.5. Data Extraction

A standard form was jointly designed by two reviewers for collecting the relevant data from each study for the following information: (1) patient characteristics; (2) trial design; (3) rTMS protocol; (4) outcome measures; (5) the duration of follow-up; and (6) mean difference and standard deviation (SD) of the scores immediately (short term) and chronically (long term) after the interventions (assessment within one day after the last rTMS session was considered as short-term outcome; assessment at one month or longer after the last rTMS session was considered long-term outcome [40]). Statistical analysis used the data of between different interventions. If the changes in scores of both groups were not clearly defined, the mean and SD of the scores after intervention for both groups were extracted on the premise of no statistical differences in baseline between the two groups. If the outcome was expressed only as a graph, the software GetData Graph Digitizer 2.25 (http://getdata-graph-digitizer.com/) was used to extract the required data.

### 2.6. Data Synthesis and Analysis

To elaborate the therapeutic effect of LF-rTMS on upper extremity recovery after stroke, the motor measures were categorized into four subclasses according to a previous study [41] of upper limb outcome measures in stroke rehabilitation: “finger flexibility,” “hand strength,” “activity dexterity,” and “body function level.” The results of the finger tapping were pooled to evaluate finger flexibility. The results of pinch force and grip force were pooled to evaluate hand strength. The results of action research arm test (ARAT), Wolf motor function test (WMFT), Jebsen-Taylor test (JTT), and nine-hole peg test (NHPT) were pooled to evaluate activity dexterity. The results of upper extremity Fugl-Meyer Assessment (FMA) were pooled to evaluate body function. For evaluating cortical excitability, the results of the rMT and MEP in both hemispheres were extracted [42, 43].

The meta-analysis was performed by using the Review Manager Software version 5.2 (Cochrane Collaboration, Oxford, England) with the formulation Hedges' *g* [44]. Data were described as mean ± SD. For the outcomes using different scales, we refer to the Cochrane Hand Book (Cochrane Collaboration, Oxford, England). The effect size of LF-rTMS was expressed by the standardized mean difference (SMD) with a 95% confidence interval (CI). The heterogeneity was tested by using the *I* ^2^ test [45]. If a significant heterogeneity was found (*I* ^2^ ≥ 50%), the random effect model was applied; otherwise, a fixed model was used. In addition, the trim and fill method [46] was constructed by using STATA/SE version 11.0 (STATA Corporation, Texas, USA) to test publication bias. The value of statistical significance was set at *P* < 0.05. Finally, effect sizes were classified as small (<0.2), medium (0.2–0.8), or large (>0.8) [47]. Sensitivity analysis was conducted to investigate the impact of lesion site, timing of stimulation from stroke onset, and other characteristics on the results.

## 3. Results

### 3.1. Study Identification

**Figure 1:**
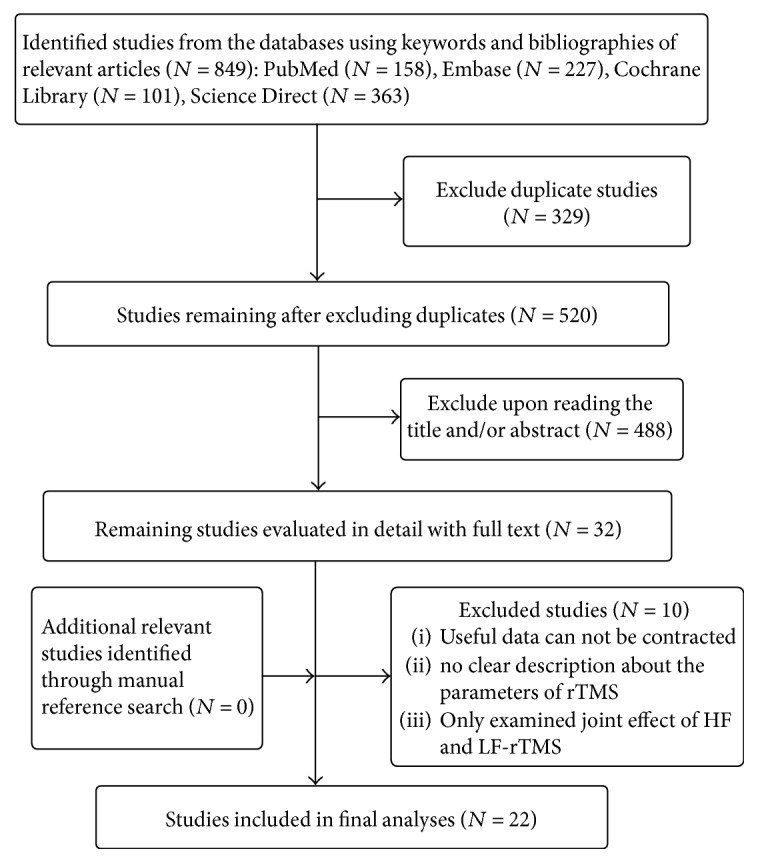
Selection process flow diagram.

Of the total 849 studies found after the initial database search, 22 studies were identified (*N* = 619) finally. The flow diagram of the selection process is shown in Figure 1.

All of the included studies applied 1 Hz rTMS over the contralesional M1. Except one study [15] that included patients with severe motor deficits and one study [21] that included patients with mild to severe deficit, all the others recruited patients with mild to moderate motor deficits. Most studies excluded the patients with other neuropsychiatric comorbidities such as aphasia, spatial neglect, or visual field deficit. Five studies [20, 24–27] used LF-rTMS as monotherapy and gained significant effect size; the others used LF-rTMS as cotherapy of active training, that is, in most of the studies, patients were also undergoing other treatments and training in both the rTMS and control groups. The details of the included studies and the results of quality assessment are shown in Tables 1 and 2 separately.

### 3.2. Motor Function Measurement

#### 3.2.1. Finger Flexibility

**Figure 2:**
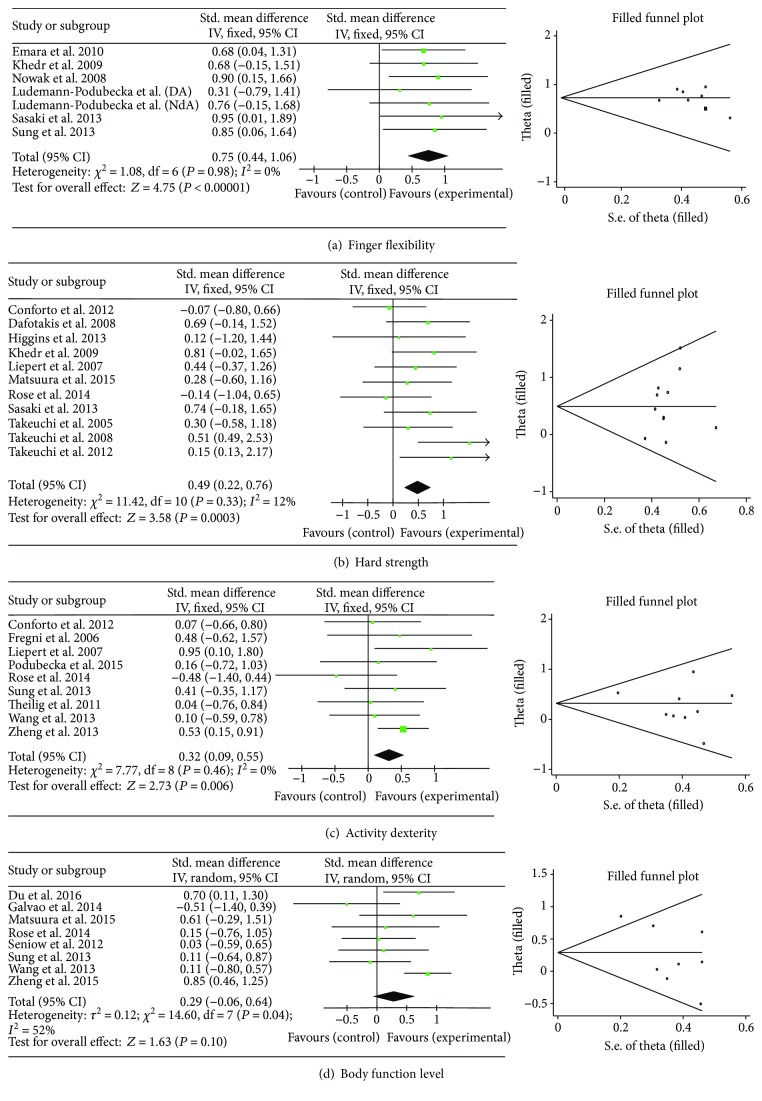
Forest plots of the short-term effect and the funnel plot analyses using the trim and fill method.

Six studies (*N* = 176) [8–10, 27–29] assessed the short-term finger flexibility. LF-rTMS had a high medium mean effect size of 0.75 (95% CI = 0.44–1.06; *P* < 0.001) without heterogeneity (*I* ^2^ = 0%) (fixed-effect model) (Figure 2(a)). The SMD for long term was 0.53 (95% CI, 0.12–0.94; *P* = 0.01) without heterogeneity (*I* ^2^ = 0%).

#### 3.2.2. Hand Strength

Eleven studies (*N* = 227) [9, 10, 17–25] evaluated short-term hand strength that showed a medium effect size of LF-rTMS therapy (SMD = 0.49; 95% CI = 0.22–0.76; *P* < 0.001; and *I* ^2^ = 12%) in the fixed-effect model (Figure 2(b)). No significant treatment effect was found for long-term effect: SMD = 0.38; 95% CI = −0.36 to 1.13; *P* = 0.31; and *I* ^2^ = 58%.

#### 3.2.3. Upper Limb Activity Dexterity

The pooled outcomes of ten trials (*N* = 299) [15, 21, 23, 24, 26, 28, 29, 32, 33] were used to evaluate the short-term upper limb activity dexterity. The result of the fixed-effect model showed a medium effect size of 0.32 (95% CI = 0.09–0.55; *P* = 0.006) without heterogeneity (*I* ^2^ = 0%) (Figure 2(c)). No significant long-term treatment effect was found: SMD = 0.14; 95% CI = −0.22 to 0.49; *P* = 0.45; and *I* ^2^ = 0%.

#### 3.2.4. Body Function Level

The pooled results from seven studies (*N* = 313) [23, 25, 28, 30–34] for short-term effect of LF-rTMS on body function level showed a nonsignificant mean effect size of 0.29 (95% CI = −0.06–0.64; *P* = 0.10) (random effect model) due to the presence of heterogeneity (*I* ^2^ = 52%) (Figure 2(d)). No significant long-term effect of LF-rTMS was found on body function [23, 30, 31]: SMD = 0.10; 95% CI = −0.70 to 0.90; *P* = 0.80; and *I* ^2^ = 77%.

#### 3.2.5. Comparison of the Motor Effect Sizes

**Figure 3:**
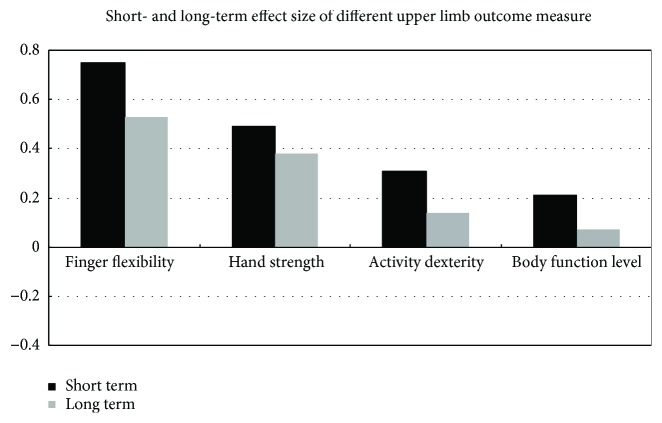
The bars show the pooled effect sizes of various upper extremity measure outcomes.

The short-term effectiveness of LF-rTMS appears to follow this descending order: finger ability is greater than hand strength which is greater than the activity dexterity and greater than body function. A similar long-term therapeutic effect of LF-rTMS was observed (Figure 3).

### 3.3. Neurophysiologic Measurement

#### 3.3.1. MEPs in Both Hemispheres

**Figure 4:**
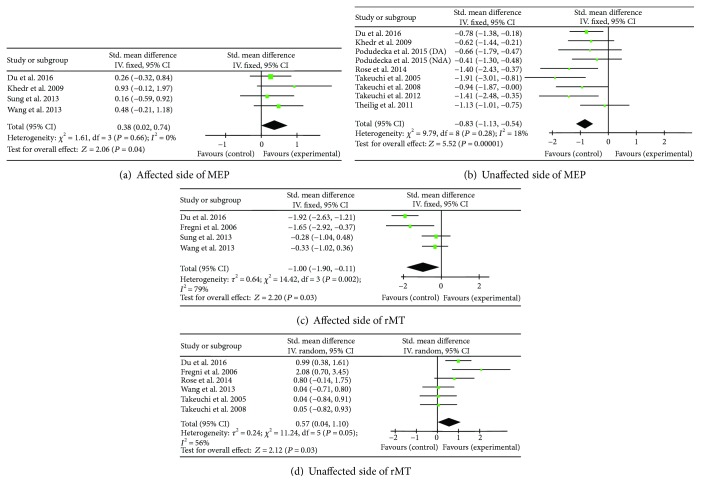
Forest plots of the mean effect sizes for MEP and rMT between the affected hand and unaffected hand. MEP: motor-evoked potential; rMT: resting motor threshold.

Four studies (*N* = 122) [10, 28, 32, 34] were pooled to explore the effects of LF-rTMS on MEPs in the affected hemisphere; and eight studies (*N* = 200) [10, 15, 17–19, 23, 29, 34] were pooled for MEPs in the unaffected hemisphere, by using the fixed effect model with the amplitude of the MEPs. The results showed a significant enhancing effect of MEP in the affected hemisphere (SMD = 0.38, 95% CI = 0.02–0.74; *P* = 0.04) without heterogeneity (*I* ^2^ = 0%) (Figure 4(a)) and a highly significant suppressing effect of MEP in the unaffected hemisphere (SMD = −0.83, 95% CI = −1.13 to −0.54; *P* < 0.0001), without significant heterogeneity (*I* ^2^ = 18%) (Figure 4(b)).

#### 3.3.2. rMTs in Both Hemispheres

Four studies (*N* = 121) [26, 28, 32, 34] assessed the effect of LF-rTMS on rMT of the affected hemisphere by using the fixed-effect model that showed a large suppressing effect size (SMD = −1.00, 95% CI = −1.90 to −0.11; *P* = 0.03; *I* ^2^ = 79%) (Figure 4(c)). LF-rTMS, however, induced an enhancing effect on rMT at a trend level in the unaffected hemisphere (SMD = 0.57; 95% CI = 0.04–1.10; *P* = 0.03; and *I* ^2^ = 56%) (Figure 4(d)).

### 3.4. Publication Bias

Funnel plots conducted with the trim and fill method for the included studies were illustrated in Figure 2. The trim and fill analyses showed that only the “finger flexibility” subclass had one study trimmed and the effect size was only slightly affected (adjusted effect size = 0.73, 0.43–1.02); no deletion or trimming occurred to other three subclasses and the effect sizes were unchanged.

### 3.5. Sensitivity Analyses

The lesion site and poststroke duration were matched between the four subgroups in two sensitivity analyses. One of the sensitivity analyses excluded eight trials that only involved subcortical stroke [17–20, 24–27] (based on the above four categories of motor function, SMD were 0.72, 0.28, 0.26, and 0.15) and the other excluded nine trials [10, 17–19, 22–24, 26, 31] that only involved acute/chronic stroke (<two weeks/>six months) [11] (SMD were 0.76, 0.36, 0.32, and 0.33), whereas the third sensitivity analysis only included rTMS plus motor training cotherapy after excluding five trials [20, 24–27] that did not specify potential cotherapy. The results were SMD = 0.72, 0.50, 0.26, and 0.15 (online-only data Supplement Figures I, II, and III).

## 4. Discussion

The present analysis provides the evidence that LF-rTMS applied over the contralesional M1 was effective for upper limb motor recovery, probably through modulating cortical excitability in poststroke patients. Although most of the trial participants were also undergoing other trainings, the trainings were carried out in both groups (rTMS group and control group) which could partially offset the impact of training on results. However, it is still not clear if the efficacy of LF-rTMS was due to its own function or its synergistic effect with other trainings. And more researches are needed in this direction.

These upper limb motor recoveries follow the previously reported four different effects of LF-rTMS on finger dexterity, hand strength, activity dexterity, and body function level [41]. Based on this classification, the short-term effectiveness of LF-rTMS appears to follow this descending order: finger ability is greater than hand strength and is greater than activity dexterity. The improvement in body function did not reach a significant level. A similar long-term therapeutic effect of LF-rTMS was observed, that is, rTMS not only produced short-term acute clinical effects but also maintained such motor improvement at the distal of the affected upper limb than at the proximal end (Figure 3).

Long-term efficacy is more important than short-term efficacy, because long-lasting beneficial effect of rTMS on upper limb motor function is a more reliable indicator for a successful clinic intervention. It is noted that although the descending trends of the various motor classifications were consistent between short term and long term—the effect size was larger at short term than at long term. Based on the follow-up data and because of the difference between the short-term and long-term effect size of LF-rTMS, it was inferred that LF-rTMS can not only produce better functional improvements but also accelerate this process in stroke patients. In other words, at short term, LF-rTMS stimulates the speed and degree of the motor recovery; whereas, at long term, LF-rTMS further maintains and improves the degree of recovery. Further research is required to test this hypothesis.

Different motor scales measured the domains differently. A better understanding of the different outcome measures and accurate interpretation of the results can help guide more efficient rehabilitation of the patient under different clinical conditions. For example, finger tapping and grip force could inform more about fine finger manipulation tasks and grasping abilities, respectively, whereas the FMA represents mixed measures, with most items (87%) related to the body structure domain [41]. Discrepancy exists in the literature. One early study showed no significant effect of LF-rTMS on upper limb coordination in motor outcomes [30]. Another study found no significant effect of LF-rTMS on the whole arm movements except for grip force [23]. Other studies, however, reported marked motor improvements of the finger and hand after LF-rTMS therapy [10, 17–20].

Although the mechanism is unknown, the results of this analysis may provide some explanations. It is known that the adaptive reorganization of stroke-induced motor deficit follows the patterns of from-the-proximal-to-distal limb and the distal limb especially the upper limb which is the most difficult to rehabilitate after stroke according to the neurodevelopment treatment [48]. The results of this meta-analysis indicate that LF-rTMS may be more effective in targeting the distal limb. One explanation for the discrepancy is that the LF-rTMS of our included trials was directed at the M1 which contributes to the high order control of motor behaviors [3]. It is known that the hand movement representation of the cortex coordinates upper limb movements through forearm muscle-controlled wrist, elbow, and shoulder [10]. Another possibility is that the speed and dexterity of finger movement are controlled primarily by corticospinal projections that are often damaged after stroke [10], but they are more readily targeted and influenced by rTMS application on the corticospinal projections. In contrast, combined activities that depend on both corticospinal and brain stem spinal pathways are less influenced by rTMS [10].

To avoid the possibility that some significant outcomes might be due to a high initial motor control, only the data of intergroup differences were analyzed. In our analysis, except one study [15] that recruited patients with severe motor deficits, all other studies recruited patients with mild-to-moderate motor deficits who did not show substantial functional disparity in both hand and arm motor outcomes. As such, our current findings may only apply to those patients of mild-to-moderate stroke. Besides, the sensitivity analysis of the trials which involved only the active training plus LF-rTMS versus those LF-rTMS without training produced similar results as the original combined results. Therefore, rTMS could indeed make further improvement on the hand flexibility which is considered the most difficult part of upper limb motor rehabilitation and which has limited success using the traditional training rehabilitation techniques alone [48].

There is evidence that cortical reorganization occurs during motor recovery of stroke [49]. The shift of balance in cortical activation between the two hemispheres has been vigorously investigated in stroke patients [3]. Compared with most other therapies, the curative effect of rTMS on stroke is based upon the activity changes of the cortex. Decreasing the excitability of corticospinal neurons, as reflected in the cumulative increase of rMT and decrease of MEP in the unaffected hemisphere, has been found associated with motor recovery [50]. However, a previous meta-analysis [36] did not show significant motor cortex improvements though a trend of positive changes in the MEP and MT groups was found. This may be due to the fact that both the LF-rTMS and HF-rTMS studies were included in the meta-analysis which included only very limited number of studies. In this current study, the LF-rTMS induced a highly significant suppressing effect on MEP in the contralesional hemisphere and a significant enhancing effect on MEP in the ipsilesional hemisphere. However, because only three trials evaluated MEP of the ipsilesional hemisphere, more studies are required to reach a reliable conclusion. A similar regulatory effect of cortical excitation exists for the results of rMT, but enhanced rMT only at a trend level in the contralesional hemisphere. These pooled effects were in agreement with the previous reports of the positive effect of LF-rTMS in modulating cortical excitability after stroke [26, 28, 32].

It is known that rTMS could enhance the motor function recovery of paretic upper limbs [51]. Increasing factors are shown to influence the effects that should be investigated in order to optimize the therapeutic effect of rTMS. A number of studies have been done in this regard. It is recognized that valid comparable measurement across studies is required to compare the effect of different interventions. So far, however, there is no consensus yet regarding the best outcome measures for evaluating hand function rehabilitation. FMA is one of the most common outcome measures used by 36% of the studies that reported hand motor rehabilitation. Santisteban et al. [41] suggested that homogenous outcome measures were critical for across study efficacy evaluation of different rehabilitation techniques and feasibility of meta-analyses that were missing in earlier assessments for upper limb motor function. This present study demonstrates that it is possible to evaluate the motor outcomes at four different levels that can specify different motor recoveries of the various parts of the upper limb following LF-rTMS.

A recent study showed that differences in patients' characters and stimulation parameters such as age, gender, lesion location, and timing from stroke onset as well as frequency of rTMS could influence the effects of rTMS on upper extremity motor recovery [51]. However, the exact stimulation parameter for different patients remains to be experimentally determined. For example, one recent study demonstrated age-dependent motor cortical plasticity in LF-rTMS-treated patients, but not in HF-rTMS-treated stroke patients [51]. Another study showed that HF-rTMS was more beneficial for motor improvement than LF-rTMS in the early phase [52], but not in the late phase of stroke [10]. Thus, the optimal protocols of rTMS for different types of upper limb rehabilitation still need to be elucidated by large cohort studies and big data analysis.

Recently, Meyer et al. [53] reported that somatosensory impairments are negatively associated with motor recovery in the upper limb. This suggests that the level of the remaining sensorimotor control may play a role in neurorehabilitation. To date, most of the published rTMS studies on motor recovery in stroke patients have not reported on sensorimotor coimpairments and most of the studies excluded patients with neuropsychiatric comorbidities such as aphasia, spatial neglect, or visual field deficit which are positively correlated with the severity of somatosensory deficits [53]. Accordingly, it may be inferred that the present results would hardly be affected by mild to moderate sensorimotor impairment, but for the more severe sensorimotor impairment, proof-of-principle studies would be necessary. In addition, consensus in outcome measurement, validation of rTMS frequency, treatment timing and duration, and lesion sites in different age groups of male and female patients could refine the current findings.

Some limitations exist in this study. First, several uncontrollable variables of the patients such as age, gender, side of onset, severity of motor deficit, and sensorimotor impairment may confound the results. Second, variations in the number of trial days (i.e., session numbers) and stimulus intensity of rTMS interventions may affect the results. Especially, the more number of rTMS trial days and increased number of pulses could be more effective [54]. Of the four functional outcome categories of this study, the “hand strength” measurement group received the least numbers of rTMS sessions and pulses. This was followed by the “finger flexibility” group. “Activity dexterity” and “body function level” groups shared similar more numbers of rTMS sessions and pulses. It is possible that the outcome differences among the four outcome groups could still exist if each group had received equal numbers of rTMS sessions and pulses. Moreover, studies published in non-English journals were not included in this analysis.

## 5. Conclusion

This meta-analysis indicates that LF-rTMS applied over the contralesional M1 has significant add-on therapeutic effect on upper limb motor dysfunction especially the functional recovery of the hand in patients with mild-moderate stroke. Future studies should verify whether cotherapy of LF-rTMS plus training will induce better hand motor rehabilitation than that of rTMS or training monotherapy.

## Figures and Tables

**Table 1:** Characteristics of the selected studies.

Study	*N* (Exp/Ctr)	Mean age	Time poststroke	Lesion site	Trial design	rTMS protocol	Outcome measurement		Follow-up	Combined training/practice
							Motor function	Neurophysiology		
Takeuchi et al. [19]	10/10	59 Y	6–60 m	Subcortical	P	1.0 Hz, 90% rMT, 1500 pulses × 1 days	Pinch force	rMT, MEP		Motor training
Fregni et al. [26]	10/5	56 Y	6–120 m	(13/15) Subcortical	P	1.0 Hz, 100% rMT, 1500 pulses × 5 days	PPT, JTT	rMT		
Liepert et al. [24]	12/12	63 Y	<2 wks	Subcortical	C	1.0 Hz, 90% rMT, 1200 pulses × 1 days	NHPT, grip force			
Takeuchi et al. [18]	10/10	62.3 Y	7–121 m	Subcortical	P	1.0 Hz, 90% rMT, 1500 pulses × 1 days	Pinch force	rMT, MEP		Motor training
Dafotakis et al. [20]	12/12	45.5 Y	1–4 m	Subcortical	C	1.0 Hz, 100% rMT, 600 pulses × 1 days	Pinch force			
Nowak et al. [27]	15/15	46 Y	1–4 m	Subcortical	C	1.0 Hz, 100% rMT, 600 pulses × 1 days	Finger tapping,			
Khedr et al. [10]	12/12	57.9 Y	1-2 wks	Nonspecified	P	1.0 Hz, 100% rMT, 900 pulses × 5 days	Finger tapping, grip force	MEP	3 m	Passive movement
Emara et al. [8]	20/20	54 Y	2–13.5 m	Nonspecified	P	1.0 Hz, 110%–120% rMT, 1500 pulses × 10 days	Finger tapping		3 m	Physical therapy
Theilig et al. [15]	12/12	61 Y	2 wks–58 m	Nonspecified	P	1.0 Hz, 100% rMT, 900 pulses × 10 days	WMFT	MEP		Extensor activity
Takeuchi et al. [17]	9/9	61.5 Y	62–71.9 m	Subcortical	P	1.0 Hz, 90% rMT, 1200 pulses × 1 days	Pinch force	MEP		Motor training
Conforto et al. [21]	15/15	55.8 Y	5–45 days	Nonspecified	P	1.0 Hz, 90% rMT, 1500 pulses × 10 days	Pinch force, JTT		1 m	Rehabilitation treatment
Seniow et al. [30]	20/20	63.4 Y	12–129 days	Nonspecified	P	1.0 Hz, 90% rMT, 1800 pulses × 15 days	FMA		3 m	Motor training
Sasaki et al. [9]	11/9	65 Y	6–29 days	Nonspecified	P	1.0 Hz, 90% rMT, 1800 pulses × 5 days	Finger tapping, grip force			Motor training
Higgins et al. [22]	6/5	66.2 Y	18–315 m	Not reported	P	1.0 Hz, 110% rMT, 1.200 pulses × 8 days	Pinch force		1 m	Task-oriented training
Sung et al. [28]	15/12	63.2 Y	3–12 m	Nonspecified	P	1.0 Hz, 90% rMT, 600 pulses × 10 days	Finger tapping, WMFT	rMT, MEP		Occupational therapy
Wang et al. [32]	17/15	62.6 Y	2–6 m	Nonspecified	P	1.0 Hz, 90% rMT, 600 pulses × 10 days	WMFT	rMT, MEP		Task-oriented training
Rose et al. [23]	11/10	64.6 Y	7–150 m	Not reported	P	1.0 Hz, 100% rMT, 1200 pulses × 16 days	Grip force, FMA	rMT, MEP	1 m	Functional task practice
Galvão et al. [31]	10/10	61 Y	>6 m	Not reported	P	1.0 Hz, 90% rMT, 1500 pulses × 10 days	FMA		1 m	Physical therapy
Ludemann-Podubecka et al. [29]	20/20	67 Y	0.25–4 m	Nonspecified	P	1.0 Hz, 100% rMT, 900 pulses × 15 days	Finger tapping, WMFT	MEP	6 m	Task-oriented training
Zheng et al. [33]	55/53	66 Y	<1 m	Nonspecified	P	1.0 Hz, 90% rMT, 1800 pulses × 24 days	FMA, WMFT			Occupational therapy
Matsuura et al. [25]	10/10	73. Y	<1 m	Subcortical	P	1.0 Hz, 100% rMT, 1200 pulses × 5 days	Grip force, FMA			
Du et al. [34]	23/23		3 days–1 m	Nonspecified	P	1.0 Hz, 110–120% rMT, 1200 pulses × 20 days	FMA		6 m	Motor exercises

Ctr: control group; Exp: experimental group; P: parallel sham control; C: crossover sham control; FMA: Fugl-Meyer assessment; ARAT: action research arm test; JTT: Jebsen-Taylor test; m: month; MEP: motor-evoked potential; NHPT: nine-hole peg test; PPT: purdue pegboard test; rMT: resting motor threshold; wk: week; Y: years; WMFT: Wolf motor function test.

**Table 2:** Quality appraisal of the selected articles.

Study	Blind process	Description of baseline data	Dropout	Point estimate and variability	Overall quality appraisal score
Takeuchi et al. [19]	2	1	0	0	3
Fregni et al. [26]	2	1	0	1	4
Liepert et al. [24]	2	0	0	1	3
Takeuchi et al. [18]	2	1	0	0	3
Dafotakis et al. [20]	0	1	0	1	2
Nowak et al. [27]	0	1	0	1	2
Khedr et al. [10]	2	1	0	1	4
Emara et al. [8]	2	1	0	1	4
Theilig et al. [15]	2	1	0	0	3
Takeuchi et al. [17]	1	1	0	1	3
Conforto et al. [21]	2	1	1	0	2
Seniow et al. [30]	2	1	7	0	2
Sasaki et al. [9]	0	1	0	0	1
Higgins et al. [22]	1	1	2	0	1
Sung et al. [28]	2	1	0	1	4
Wang et al. [32]	2	1	0	1	4
Rose et al. [23]	2	1	3	0	2
Galvão et al. [31]	2	1	0	0	3
Ludemann-Podubecka et al. [29]	2	1	0	0	3
Zheng et al. [33]	2	1	4	0	2
Matsuura et al. [25]	2	1	0	1	4
Du et al. [34]	2	1	0	1	4

In the case of any dropout, the total score will be subtracted by 1.

## Supplementary Materials

Supplementary Materials Supplementary Figure I: sensitivity analysis examining whether the result was influenced by lesion site. Supplementary Figure II: sensitivity analysis examining whether the result was influenced by combining training. Supplementary Figure III: sensitivity analysis examining whether the result was influenced by time post stroke.
