# Supplementary material for: Low-Frequency Repetitive Transcranial Magnetic Stimulation for Stroke-Induced Upper Limb Motor Deficit: A Meta-Analysis
Source: Neural Plast. 2017 Dec 21;2017:2758097. doi: 10.1155/2017/2758097 (PMC5756908; doi:10.1155/2017/2758097)
Supplement: Supplementary Materials — Supplementary Figure I: sensitivity analysis examining whether the result was influenced by lesion site. Supplementary Figure II: sensitivity analysis examining whether the result was influenced by combining training. Supplementary Figure III: sensitivity analysis examining whether the result was influenced by time post stroke. [file 2758097.f1.doc]

SUPPLEMENTAL MATERIAL


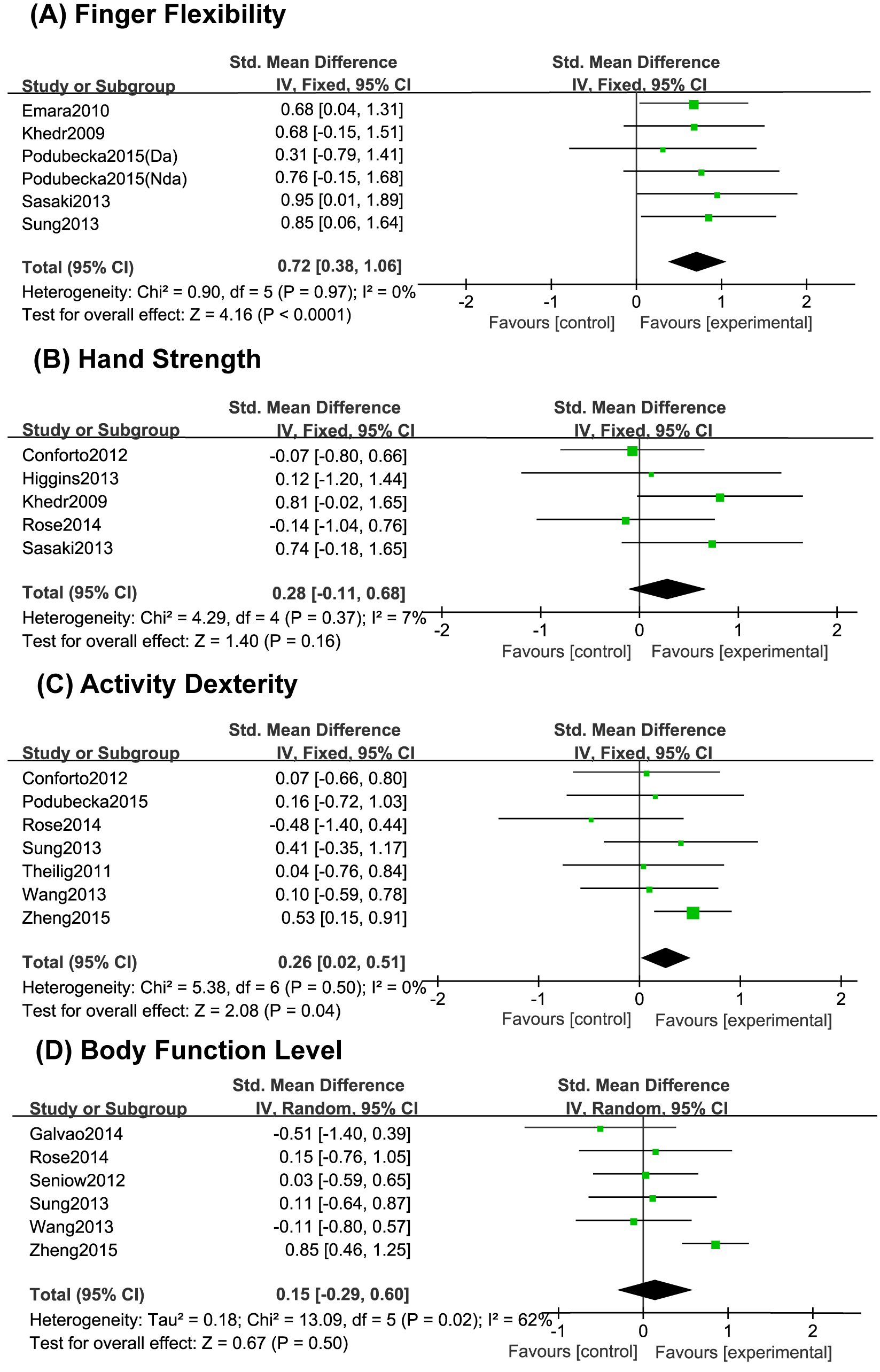


Supplementary Figure I. Sensitivity analysis examing whether the result was influenced by lesion site.


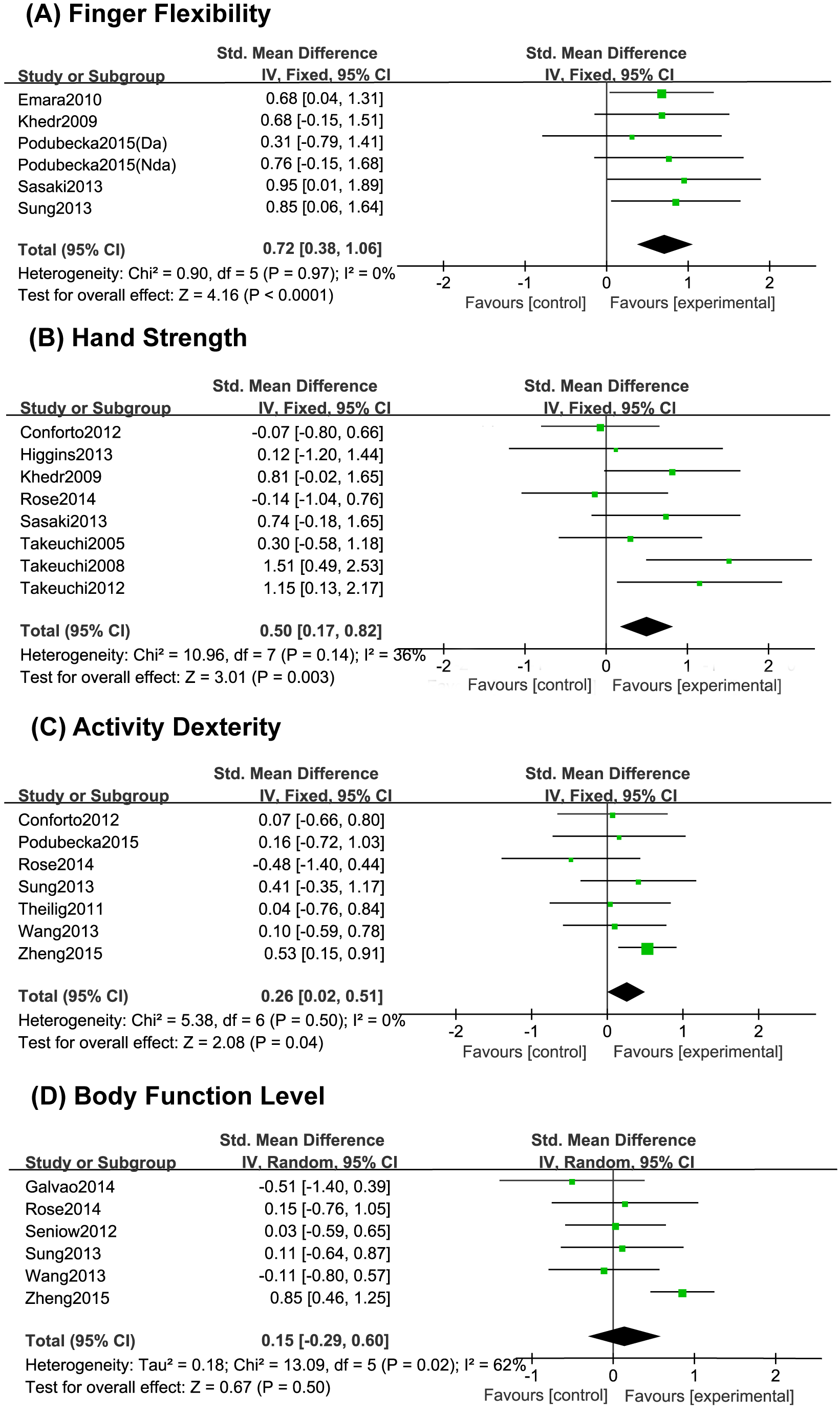


Supplementary Figure II. Sensitivity analysis examing whether the result was

influenced by whether combing training.


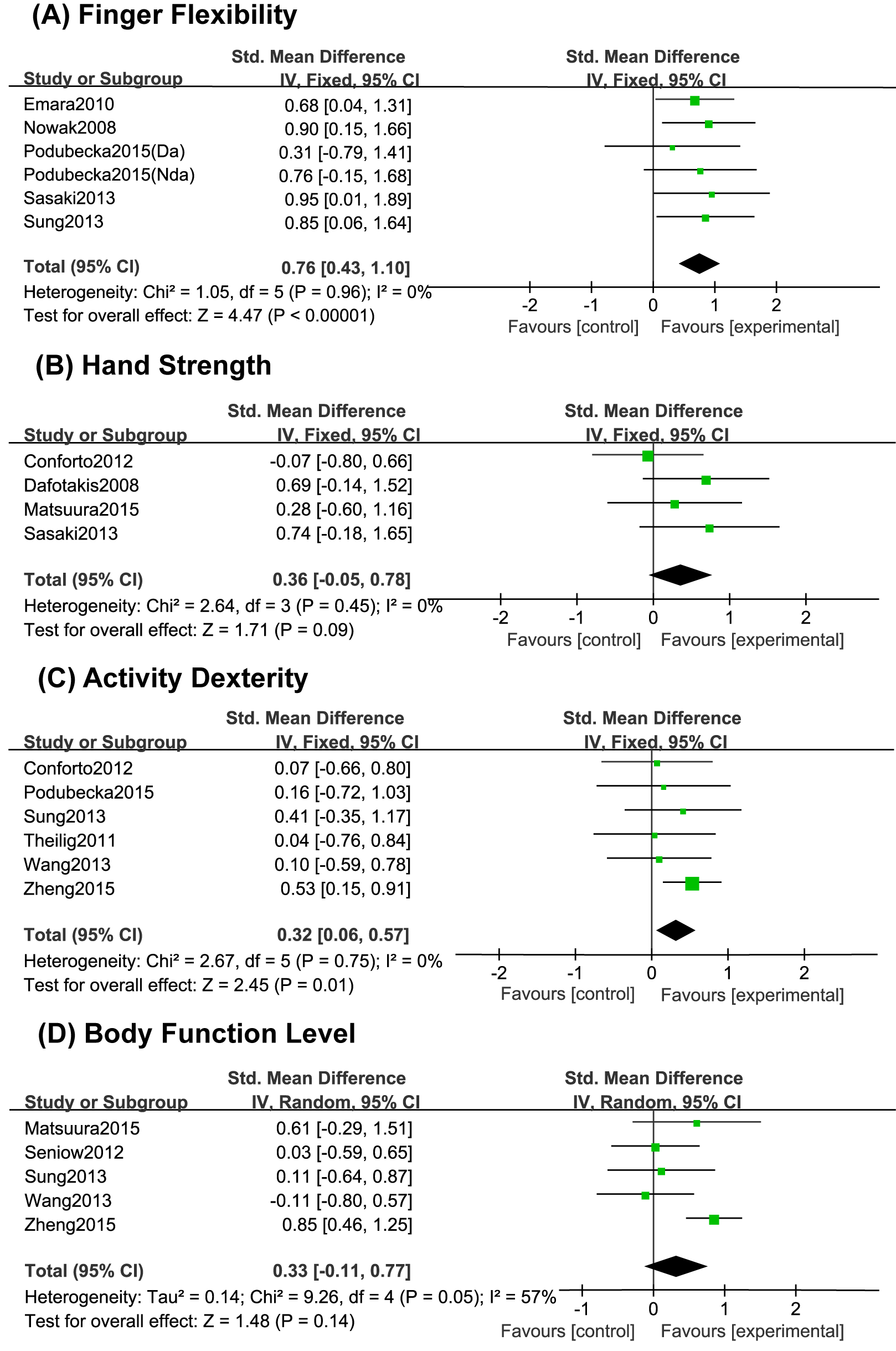


Supplementary Figure III. Sensitivity analysis examing whether the result was

influenced by time post-stroke.
